# Bile cell-free DNA shows benefit as a potential sample for somatic mutation detection of resectable cholangiocarcinoma

**DOI:** 10.1016/j.iliver.2025.100206

**Published:** 2025-11-07

**Authors:** Songyao Liu, Xiaowu Ma, Hongkai Zhuang, Bingkun Wang, Qingbin Wang, Yonglin Hua, Ziyu Zhang, Yuxin Xiao, Jianbo Wan, Yajin Chen, Changzhen Shang

**Affiliations:** a Department of Hepatobiliary Surgery, Sun Yat-sen Memorial Hospital, Sun Yat-sen University, Guangzhou 510080, Guangdong, China; b Guangdong Provincial Key Laboratory of Malignant Tumor Epigenetics and Gene Regulation, Sun Yat-sen Memorial Hospital, Sun Yat-Sen University, Guangzhou 510080, Guangdong, China; c Department of Hepatobiliary and Pancreatic Surgery, Qunli Branch, The First Affiliated Hospital of Harbin Medical University, Harbin 150010, Heilongjiang, China; d State Key Laboratory of Mechanism and Quality of Chinese Medicine, Institute of Chinese Medical Sciences, University of Macau, Taipa 999078, Macau

**Keywords:** Hepatobiliary cancers, Liquid biopsy, Next-generation sequencing, Single-nucleotide variant, Tumor mutation burden

## Abstract

**Background and aims:**

Cholangiocarcinoma (CCA), a highly aggressive hepatobiliary malignancy, faces significant diagnostic challenges in early stages due to nonspecific clinical manifestations. Although plasma cell-free DNA (cfDNA) analysis via next-generation sequencing (NGS) is widely used for noninvasive cancer detection, its sensitivity in CCA remains suboptimal owing to the low tumor DNA abundance. This study aims to compare bile and plasma as sources for liquid biopsy in CCA genomic profiling, focusing on analytical accuracy and biomolecular stability.

**Methods:**

Ten patients with resectable CCA were prospectively enrolled in this study. DNA extracted from matched plasma, bile, and tumor tissue underwent NGS for somatic mutation, microsatellite stability (MSI), and tumor mutation burden (TMB) analysis.

**Results:**

The concentration of cfDNA in bile was significantly higher than in plasma. Somatic mutations were detected in 100% (10/10) of tumor tissue DNA, 70% (7/10) of bile cfDNA, and 10% (1/10) of plasma cfDNA. Compared to plasma cfDNA, bile cfDNA exhibited higher TMB (median TMB 1.0 vs. 0; *p* < 0.05) and greater maximum allele fractions (median maxAF 0.057 vs. 0; *p* < 0.001). While tumor stage significantly influenced plasma cfDNA detection rates, bile cfDNA maintained high detection sensitivity independent of clinical variables. Among CCA patients, bile cfDNA mutation positivity demonstrated sensitivity comparable to CA19-9 serology.

**Conclusion:**

Bile cfDNA outperforms plasma cfDNA in detecting somatic mutations and retains high sensitivity independent of tumor stage, positioning it as a promising tool for early CCA diagnosis and molecular characterization.

## 1 Introduction

As a malignancy derived from the biliary epithelium, cholangiocarcinoma (CCA) exhibits marked invasiveness and stromal desmoplasia. Based on anatomical location, it is classified into intrahepatic (iCCA) and extrahepatic (eCCA) subtypes. The extrahepatic variant can be further subdivided into perihilar (pCCA) and distal (dCCA) types according to the confluence point of the cystic duct and hepatic duct.^1^^,^^2^ In Asia, the incidence of CCA is increasing and notably higher than in Europe and America.^3^^,^^4^ Despite its rarity, CCA carries a high mortality rate due to the subtlety of early symptoms. The 5-year survival rates are only 19.9% for eCCA and 10.8% for iCCA.^5^ Meanwhile, CCA exhibits high tumor heterogeneity, which is affected by anatomical location, histological characteristics, and cell origin.^6^ These characteristics not only limit the effectiveness of traditional therapies like chemotherapy but also underscore the critical need for early detection and precision treatment approaches.^7^^,^^8^

While histopathological specimens maintain their status as the gold standard for molecular oncology assays, the anatomical complexity of biliary tumors often makes direct tissue acquisition risky, with potential complications including bleeding and pancreatitis unless obtained through surgical resection.^9^ As the least invasive alternative, liquid biopsy has emerged as a valuable tool for clinical mutation detection, dynamic monitoring, and tumor diagnosis across various malignancies.^10^ Plasma cfDNA testing is the most widely adopted liquid biopsy method. However, it still faces limitations in terms of low detection rates in some tumors, particularly the early stage of pancreatic cancer and CCA due to anatomical barriers. Previous studies have reported that various body fluid specimens, including pleural fluid, cerebrospinal fluid, sputum, ascites, and urine may outperform plasma in assessing tumor progression and resistance mechanisms for specific cancers.^11^, ^12^, ^13^, ^14^, ^15^ As a hepatobiliary system-specific bodily fluid, bile can be readily obtained through endoscopic nasobiliary drainage (ENBD) and percutaneous transhepatic cholangiography drainage (PTCD), making it a promising candidate for advanced molecular detection methods in CCA.

Bile has been proposed as an optimal sample for cfDNA testing due to its high concordance with tissue DNA and ability to capture tumor-specific molecular features more effectively than plasma cfDNA.^16^ The mutation detection rates (MDR) vary significantly depending on the subtype and stage of CCA.^17^ Additionally, bile-derived cfDNA demonstrated higher consistency with tissue samples across multiple mutation dimensions.^18^ Despite these advantages, current research remains limited by small cohort sizes and frequent lack of matched tumor tissue controls. Several key questions regarding bile cfDNA analysis in CCA remain unresolved, including its utility for identifying immunotherapy biomarkers, such as tumor mutational burden (TMB) and microsatellite instability (MSI). There exists an urgent need for larger-scale studies to evaluate bile cfDNA's clinical applications in CCA management comprehensively.

This study proposes to address these gaps by enrolling ten resectable CCA patients and performing comprehensive genomic profiling of matched tumor tissue, plasma cfDNA, and bile cfDNA samples using a 520-gene panel NGS assay. Building upon prior research, we aim to validate and further investigate CCA-related biomarkers (including TMB, MSI, and homologous recombination deficiency [HRD] scores) while analyzing potential correlations among these parameters. Our ultimate objective is to provide robust evidence supporting precision medicine strategies for CCA patient care.

## 2 Methods

### 2.1 Patient selection

Between February 2023 and February 2024, a prospective cohort of 10 eligible patients with resectable CCA underwent screening based on the following inclusion criteria: (1) with CCA diagnosis confirmed by pathological examinations; (2) with Eastern Cooperative Oncology Group (ECOG) score ≤2; (3) without other malignant tumors. The study was approved by the Ethics Committee of Sun Yat-sen Memorial Hospital, Sun Yat-sen University. Informed consent was obtained from each patient for the use of their tumor, peripheral blood, and bile samples.

### 2.2 Sample collection

Matched tumor tissue, venous blood, and bile specimens were collected from CCA patients. Surgical resection-derived tissues underwent standard fixation in 10% neutral-buffered formalin followed by paraffin. Venous blood (10 mL per patient) was collected in K2-EDTA tubes and subjected to biphasic centrifugation (1800 × *g*, 10 min, 4 °C) to separate plasma from buffy coats. Plasma was aliquoted for subsequent cfDNA isolation, while the WBC sediments served as matched germline controls for genomic DNA extraction. Bile samples (10 mL) obtained via PTCD or ENBD were collected and processed within 2 h post-collection. Primary centrifugation (1800 × *g*, 15 min, 4 °C) removed cellular components, followed by secondary clarification centrifugation (1800 × *g*, 10 min, 4 °C) of the supernatant. The final supernatant underwent optical validation via light microscopy to confirm the absence of residual cells or debris, with aliquots cryopreserved at −80 °C pending cfDNA extraction.

### 2.3 DNA extraction and NGS sequencing

Genomic DNA from tissue specimens was isolated using the QIAamp DNA FFPE Tissue Kit (Qiagen, Hilden, Germany), following the manufacturer-supplied protocols. Concurrently, cfDNA from matched plasma and bile samples underwent concurrent cfDNA isolation using the QIAamp Circulating Nucleic Acid Kit (Qiagen, Valencia, CA, USA), adhering to manufacturer-specified protocols for dual-source liquid biopsy analysis. Fragmentation of both tissue-derived DNA and cfDNA was performed using a Covaris M220 system (Covaris, MA, USA), with subsequent end-repair, 5′-phosphorylation, and adapter ligation steps standardized across samples. DNA fragments spanning 200–400 bp were selectively purified via the Agencourt AMPure XP Kit (Beckman Coulter, CA, USA), followed by hybridization capture using panel-specific probe baits. Magnetic bead-based hybrid selection was then employed to enrich target regions, and amplification of captured libraries was achieved through PCR. Fragment size distribution and library quality were assessed using the Qubit 2.0 fluorometer (Life Technologies, Carlsbad, CA) with the dsDNA High-Sensitivity Assay Kit. Indexed libraries were subjected to paired-end sequencing on the Illumina NextSeq 500 platform (Illumina, Inc., USA) with an average depth of 1000× . The customized panel (OncoScreen Plus, Burning Rock Biotech, Guangzhou, China) targeted 520 cancer-associated genes, encompassing 1.64 megabases of the human genome.

### 2.4 Sequencing data pretreatment

Raw sequencing data were subjected to adapter trimming and low-quality read removal using Trimmomatic (v0.36) under default parameters. Preprocessed reads were then aligned to the human reference genome (GRCh37/hg19) employing the Burrows-Wheeler Aligner (v0.7.10), with paired-end mapping strategies applied to optimize positional accuracy. Somatic variant identification was performed through comparative analysis of plasma/bile-derived sequences against matched white blood cell (WBC) controls. Initial variant calling was executed using a consensus approach integrating GATK HaplotypeCaller (v3.2), MuTect, and VarScan. Public population databases (ExAC, 1000 Genomes, dbSNP, ESP6500SI–V2) were leveraged to exclude polymorphisms with minor allele frequency (MAF) exceeding 0.1%, ensuring prioritization of rare variants. The retained variants underwent functional annotation via ANNOVAR and SnpEff (v3.6). Copy number variations (CNVs) were inferred from depth-of-coverage profiles across capture intervals using an in-house non-supervised clustering algorithm, which normalized coverage biases through singular value decomposition (SVD).^19^ Translocations were detected by integrative analysis of discordant read pairs and split-read signatures, combining TopHat2 (fusion-aware mode) and Factera (v1.4.3) outputs to minimize false positives. Allele frequencies of confirmed somatic mutations were quantified from aligned read counts supporting mutant alleles. Microsatellite instability (MSI) status was determined using a read-distribution method to compare microsatellite loci depth distributions against a validated baseline panel.^20^^,^^21^

### 2.5 Statistical analysis

Inter-group comparisons were executed utilizing Student's *t-*test for continuous variables with normal distribution or Mann–Whitney *U* test for continuous variables with non-normal distribution. Bivariate correlations were analyzed with Pearson's correlation coefficient, with linearity and normality assumptions verified. Statistical significance was defined as *p* < 0.05 for all inferential tests. Analytic workflows were conducted in R software 3.6.2 (Vienna, Austria).

## 3 Results

### 3.1 Clinicopathological characteristics of patients

The study enrolled a cohort of 10 pathologically confirmed resectable CCA patients with ECOG performance status ≤2 (Table 1). The median age of the cohort was 59.5 years, comprising 3 intrahepatic CCA (iCCA), 2 distal CCA (dCCA), and 5 perihilar CCA (pCCA) cases. Tumor staging analysis distributed the patients as follows: 1 patient with AJCC stage I disease, 3 patients with stage II (including 1 lymph node-positive case), and 3 patients with stage III (including 2 lymph node-positive cases). All patients tested negative for hepatitis B virus (HBV) infection, while 2 patients had a history of cholelithiasis. Among these patients, 9 patients suffered from obstructive jaundice. According to the Child-Pugh classification, 5 patients were graded as class A and 5 as class B. The median liver function markers of these patients were ALT 158.5 U/L and AST 120.5 U/L. Plasma tumor biomarker profiling demonstrated AFP positivity in 2 cases, elevated CEA (>7 U/L) in 3 cases, CA125 (>35 U/L) in 1 case, CA19-9 (>37 U/L) in 8 cases, and CA72-4 (>7 U/L) in 1 case, highlighting the heterogeneity of biomarker expression in this resectable CCA population.

**Table 1:** The clinical characteristics of CCA patients.

Characteristics	*n* = 10, (%)
**Age, median (range)**	59.5 (51–73)
**Gender**	
Female	4 (40.0)
Male	6 (60.0)
**Smoking**	
No	7 (70.0)
Yes	3 (30.0)
**Drinking**	
No	8 (80.0)
Yes	2 (20.0)
**Obstructive jaundice**	
No	1 (10.0)
Yes	9 (90.0)
**Cholelithiasis**	
No	8 (80.0)
Yes	2 (20.0)
**Child-Pugh**	
A	5 (50.0)
B	5 (50.0)
**CCA classification**	
ICC	3 (30.0)
DCC	2 (20.0)
PCC	5 (50.0)
**AJCC stage**	
Stage Ⅰ	1 (10.0)
Stage Ⅱ	7 (70.0)
Stage Ⅲ	2 (20.0)
**CEA (ng/mL)**	
<5	7 (70.0)
≥ 5	3 (30.0)
**CA19**-**9 (U/mL)**	
<37	2 (20.0)
≥ 37	8 (80.0)
**AFP (ng/mL)**	
≤7	8 (80.0)
>7	2 (20.0)
**CA125 (U/mL)**	
<35	9 (90.0)
≥ 35	1 (10.0)
**CA72**-**4 (U/mL)**	
≤ 7	9 (90.0)
>7	1 (10.0)
**Liver function markers, median (range)**	
ALT (U/L)	158.5 (54–889)
AST (U/L)	120.5 (40–358)
GGT (U/L)	796 (57–1541)
**Differentiation**	
High-medium	2 (20.0)
Medium	5 (50.0)
Medium-low	2 (20.0)

CCA: Cholangiocarcinoma; AJCC: American Joint Committee on Cancer; CEA: carcinoembryonic antigen; CA19-9: cancer antigen 19-9; AFP: alpha-fetoprotein; CA 125: carbohydrate antigen 125; CA72-4: carbohydrate antigen 72-4; ALT: alanine transaminase; AST: aspartate transaminase; GGT: γ-glutamyl transpeptidase.

### 3.2 cfDNA content of the bile or plasma

**Fig. 1:**
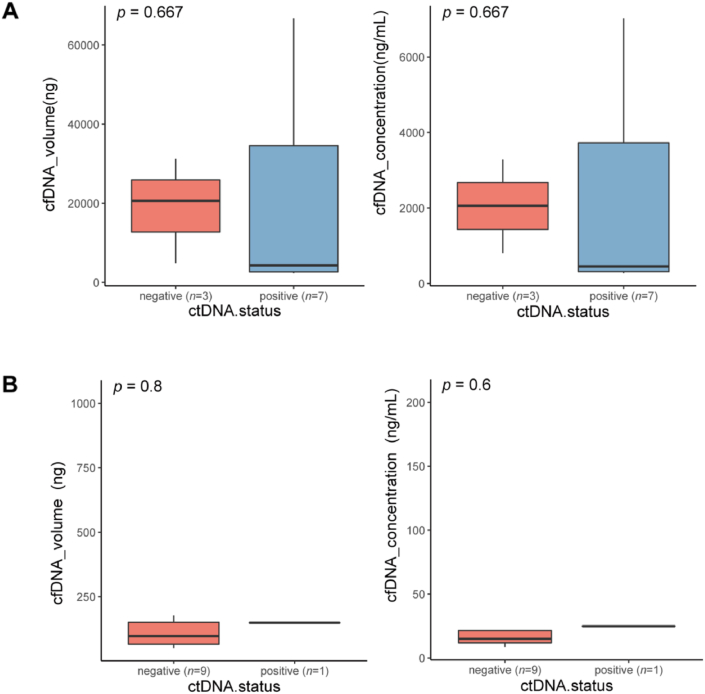
(A) Bile cfDNA somatic mutation detection result was independent of the amount of cfDNA volume as well as concentration. (B) Plasma cfDNA somatic mutation detection result was independent of the amount of cfDNA volume as well as concentration. cfDNA, cell-free DNA.

All cfDNA samples underwent a quality control process to ensure suitability for subsequent NGS analysis. Under the same conditions of extracting 10 mL samples for extraction, bile samples had an extremely higher cfDNA content than plasma, with a median cfDNA concentration of 1433.3 ng/mL versus 17.1 ng/mL (*p* < 0.001). Meanwhile, the detection results of bile and plasma cfDNA were found to be independent of the volume and concentration of cfDNA (Fig. 1).

### 3.3 Overall mutation profile of tumor DNA, plasma cfDNA, and bile cfDNA

**Fig. 2:**
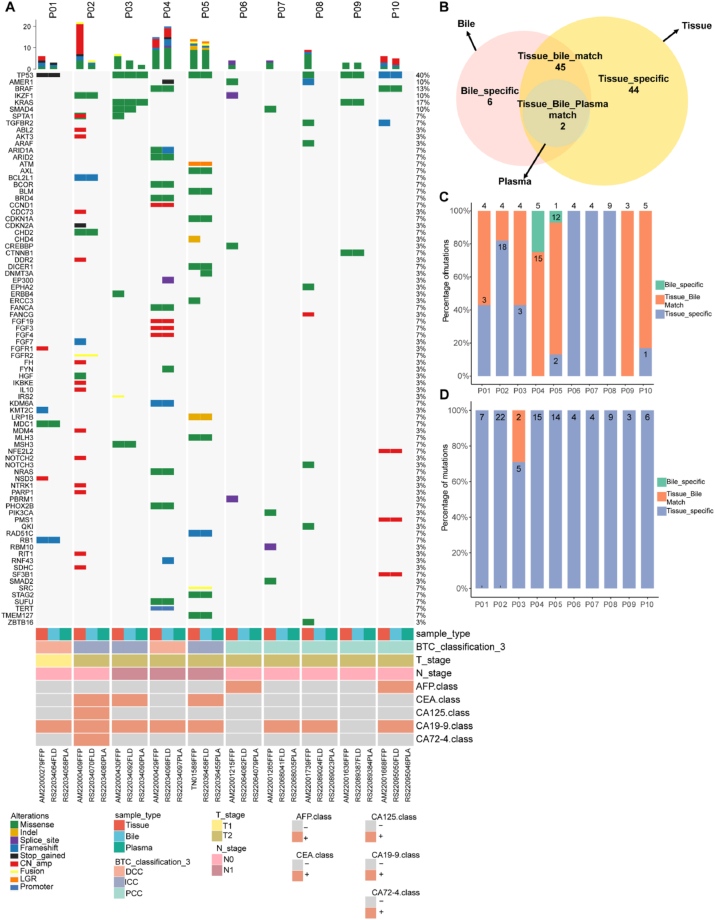
(A) Somatic mutation heatmap of tissue DNA, plasma cfDNA, and bile cfDNA. (B) Specific variants detected in bile, plasma, and tissue. (C) Mutation detected in bile cfDNA and tissue samples. (D) Mutation detected in plasma cfDNA and tissue samples. cfDNA, cell-free DNA.

This study employed a 520-panel NGS approach to analyze tumor DNA, plasma cfDNA, and bile cfDNA from 10 CCA patients, generating a comprehensive mutational profile. Across the 10 tissue samples, 103 variants were identified, comprising 61 single-nucleotide variants (SNVs), 1 inversion (INV), 14 insertions/deletions (indels), 24 copy number variations (CNVs), 1 deletion (DEL), and 2 fusions (Fig. 2A). Tumor protein 53 (*TP53*) emerged as the most frequently mutated gene, with mutations detected in 6/10 tissue samples, 1/10 plasma samples, and 5/10 bile samples. Among the bile samples, 53 variants were detected, including 34 SNVs, 9 indels, 7 CNVs, 1 DEL, and 2 fusions. Notably, 47 variants were concordant between tissue and bile samples, whereas only 1 plasma sample exhibited positive variants, with 2 SNVs confirmed in both tissue and bile. Comparative analysis of the 7 patients positive for both tissue and bile variants revealed 27 tissue-specific variants, 6 bile-specific variants, and no plasma-specific variants (Fig. 2B). Further examination of the 7 bile somatic mutation-positive samples identified 5 bile-specific mutations in sample P04 and 1 in sample P05 (Fig. 2C). In contrast, plasma cfDNA somatic mutations were detectable solely in sample P03, with no plasma-specific mutations observed (Fig. 2D).

These findings underscore the heterogeneity of mutational profiles in CCA and emphasize the critical role of integrating tissue and bile cfDNA analysis to capture the full spectrum of genetic alterations, as plasma cfDNA demonstrated limited sensitivity in variant detection. The data collectively highlight bile cfDNA as a robust complementary liquid biopsy medium for CCA molecular profiling, offering insights that may guide therapeutic strategies and biomarker development.

### 3.4 The overview of somatic mutation in tissue, plasma, and bile samples

**Fig. 3:**
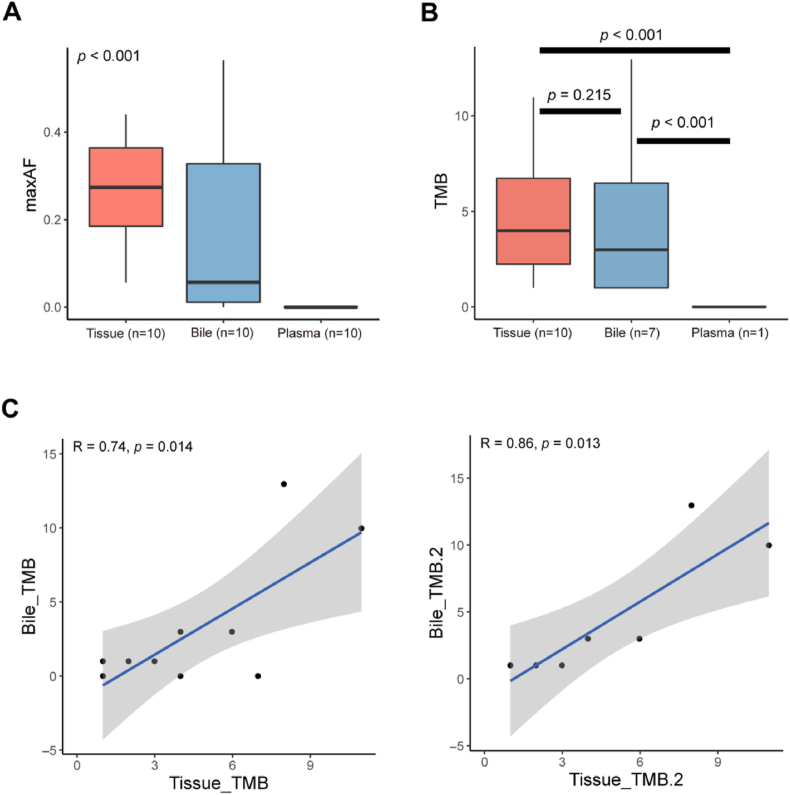
(A) Bile cfDNA showed a higher maxAFs level than plasma cfDNA. (B) Tissue and bile samples did not show a significant difference but showed significantly higher (*p* < 0.001) TMB levels than plasma, with a median level of 4.0, 1.0, and 0. (C) Bile samples showed high consistency with tissue, with R = 0.74 for the total 10 samples, and R = 0.86 for 7 bile positive samples. cfDNA, cell-free DNA; maxAFs, maximum allele fractions; TMB, tumor mutation burden.

Using tissue DNA mutation profiles as the reference standard for somatic mutations, bile samples demonstrated a significantly higher detection rate (70%) compared to plasma samples (10%). None of the tissue, plasma, or bile samples tested positive for microsatellite instability (MSI), confirming the absence of MSI in this cohort. The median maximum allele fraction (maxAF) levels in tumor samples correlated with the percentage of tumor cells in histological sections, whereas cfDNA maxAF levels reflected tumor burden. Notably, bile cfDNA exhibited markedly higher maxAF levels than plasma cfDNA (median maxAF: 0.057 vs. 0; *p* < 0.001; Fig. 3A). Tumor mutational burden (TMB) analysis revealed no significant difference between tissue and bile samples (median TMB: 4.0 vs. 1.0), but both were significantly higher (*p* < 0.001) than plasma TMB (median: 0; Fig. 3B). Furthermore, bile samples showed strong concordance with tissue samples, with a correlation coefficient (R) of 0.74 across all 10 samples and 0.86 in the 7 bile-positive cases (Fig. 3C). These findings demonstrate that bile cfDNA exhibits superior sensitivity for somatic variant detection compared to plasma cfDNA, while maintaining high concordance with tumor tissue genomic profiles. This supports the utility of bile cfDNA as a robust liquid biopsy modality for comprehensive molecular characterization of cholangiocarcinoma.

### 3.5 Correlation between cfDNA mutation detection rate (MDR) and clinical factors

**Fig. 4:**
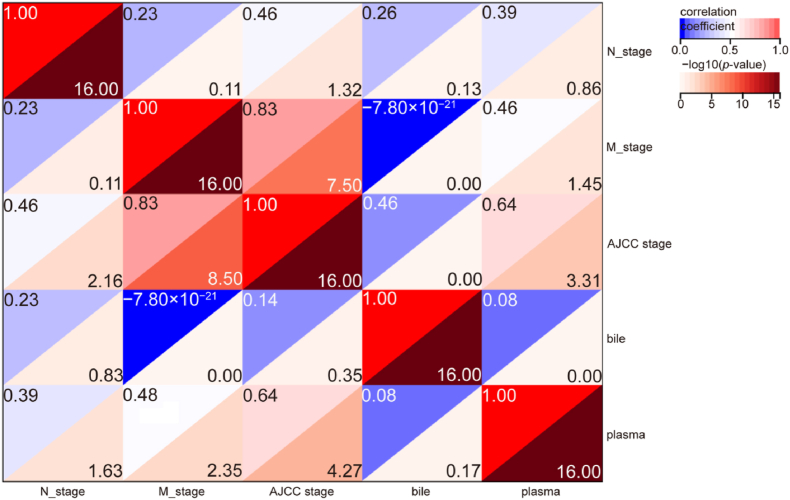
Correlations between cfDNA MDR in bile or plasma and clinical features, including AJCC stage, lymph node metastasis (N stage), and distal metastasis (M stage). cfDNA, cell-free DNA; MDR, mutation detection rate; AJCC: American Joint Committee on Cancer.

This study further investigated clinical factors potentially influencing the MDR of cfDNA. Analysis integrating published data with the current cohort (28 published cases and 10 new cases) revealed a significant positive correlation between plasma cfDNA MDR and AJCC stage (R = 0.64, *p* < 0.001). In contrast, bile cfDNA MDR demonstrated no statistically significant association with AJCC stage, lymph node metastasis, or distant metastasis (Fig. 4). To identify determinants of bile cfDNA MDR, we evaluated correlations with the following clinicopathological parameters: primary tumor location (iCCA/dCCA/pCCA), histological differentiation grade, age, sex, smoking/alcohol history, AST and ALT levels, and Child-Pugh classification. None of these factors showed a significant association with bile cfDNA MDR in the study cohort (Table 2). These findings suggest that bile-derived cfDNA mutation detection rates are largely independent of conventional clinical and tumor-related variables in CCA. Further mechanistic studies are warranted to elucidate biological drivers of interpatient variability in bile cfDNA yield and mutational representation.

**Table 2:** Relationship between MDR and clinicopathological characteristics in CCA patients.

Characteristics		Bile MDR			*p*
		total (*n* = 38)	detected (*n* = 27)	not detected (*n* = 11)	
Primary tumor location	eCCA	27	18	9	1.000
	iCCA	11	9	2	
Age	<60	14	9	5	0.993
	≥60	24	18	6	
Sex	Female	15	11	4	0.993
	Male	23	4	7	
AST (U/L)	≤40	8	7	7	0.995
	>40	30	20	10	
ALT (U/L)	≤35	7	6	1	0.995
	>35	31	21	10	
Child-Pugh	A	8	6	2	0.993
	B	30	21	9	
Histological Diffentiation grade	1	4	3	1	0.120
	2	12	8	4	
	3	7	6	1	
	4	15	10	5	
Smoking and/or Drinking	0	19	13	6	0.992
	1	19	14	5	

MDR: mutation detection rates; CCA: cholangiocarcinoma; eCCA: extrahepatic cholangiocarcinoma; iCCA: intrahepatic cholangiocarcinoma; ALT: alanine transaminase; AST: aspartate transaminase.

### 3.6 Bile cfDNA was more consistent with tumor DNA in detecting mutation profiles than plasma

**Fig. 5:**
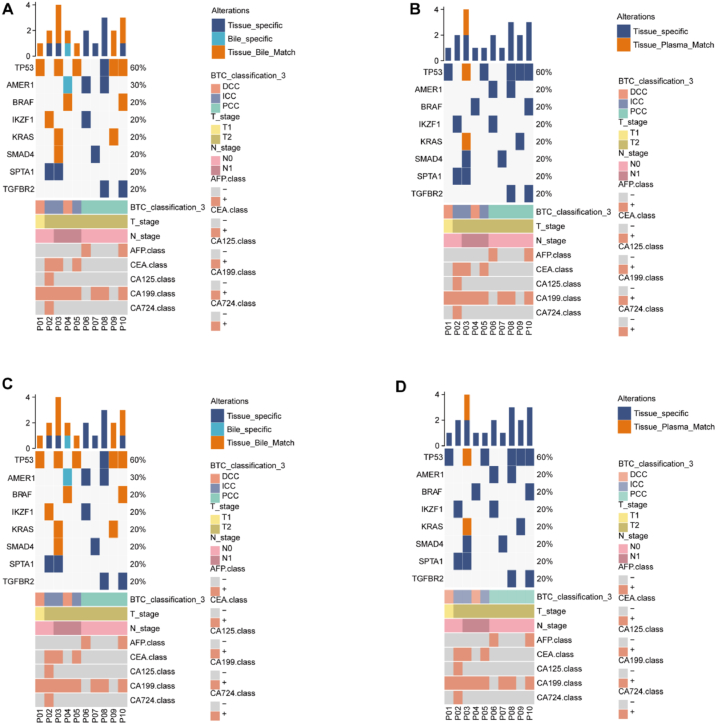
(A) Bile cfDNA sensitivities when all mutations were included. (B) Plasma cfDNA sensitivities when all mutations were included. (C) Bile and plasma cfDNA sensitivities when only SNV/INDELs were included. (D) Plasma cfDNA sensitivities when only SNV/INDELs were included. cfDNA, cell-free DNA; SNV, single-nucleotide variants; INDEL, insertions/deletions.

Somatic mutations were detected in all 10 tissue samples, establishing tumor tissue as the reference standard for subsequent analyses. When all somatic mutation types were included, higher sensitivity was demonstrated in bile-derived cfDNA compared to plasma cfDNA. The sensitivity and positive predictive value (PPV) of bile cfDNA were 51.7% and 90.2%, respectively, whereas plasma cfDNA yielded a sensitivity of 2.2% with a PPV of 100%. Among the 7 tissue-bile concordant positive cases, bile cfDNA exhibited further elevated sensitivity (63.9%) while maintaining a PPV of 90.2%. In contrast, the single tissue-plasma concordant positive case retained low plasma sensitivity (28.6%) despite 100% PPV. When analysis was restricted to SNV/InDel mutations, bile cfDNA maintained superior performance across all 10 samples (sensitivity: 58.1%; PPV: 87.8%), while plasma cfDNA remained limited (sensitivity: 3.2%; PPV: 100%). Notably, among the 7 tissue-bile concordant pairs, SNV/InDel detection achieved a sensitivity of 78.3% and a PPV of 87.8%. In contrast, the single tissue-plasma concordant pair exhibited a sensitivity of 33.3% with 100% PPV (Fig. 5).

These findings collectively indicate that bile cfDNA achieves significantly higher sensitivity than plasma cfDNA for detecting somatic mutations, including SNV/InDel variants. The stronger concordance between bile and tissue samples in mutation profiling supports the utility of bile as a robust liquid biopsy source for comprehensive genomic analysis in CCA.

### 3.7 Bile cfDNA showed a similar positive rate to traditional CCA biomarkers

**Fig. 6:**
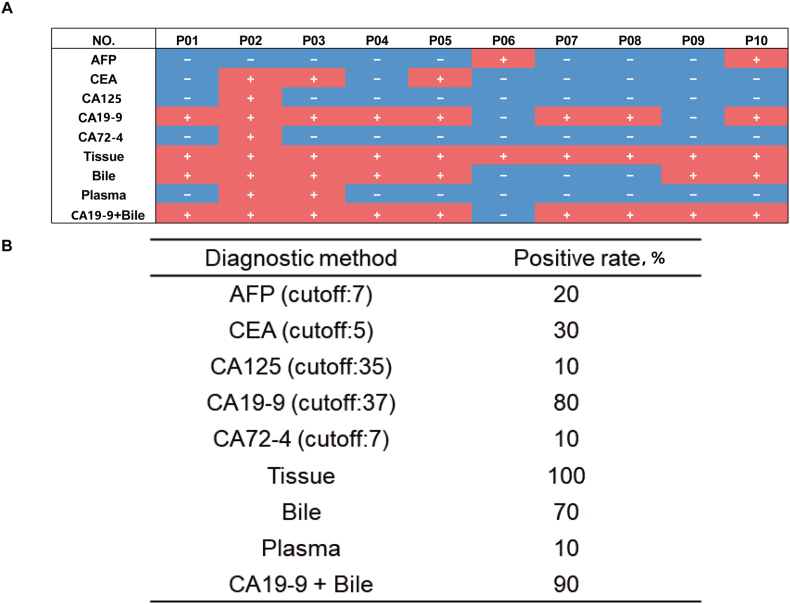
(A) Traditional CCA diagnostic basis, commonly used biomarkers, and cfDNA results were involved to establish a diagnostic model. Patients with mutations detected in tissue, bile, and plasma were marked as "+" and those with a completely negative mutation spectrum were marked as "−". (B) Positive rate of each diagnostic method. CCA, cholangiocarcinoma; cfDNA, cell-free DNA.

A comparative analysis was conducted between the positive rates of conventional clinical biomarkers and somatic mutation detection in cfDNA to further evaluate diagnostic consistency. Among standard biomarkers, the highest standalone positive rate was observed for CA19-9 (80%), while bile cfDNA exhibited a positive rate of 70%, substantially exceeding that of plasma cfDNA. When CA19-9 was combined with bile cfDNA somatic mutation analysis, the positive rate was further elevated to 90% (Fig. 6). These results suggest that the integration of CA19-9 with bile cfDNA-based mutation profiling may establish a potential diagnostic foundation for unresectable CCA.

## 4 Discussion

Through comprehensive analysis of matched tissue, plasma, and bile samples from patients with resectable CCA, our study evaluated the capability of bile cfDNA to detect somatic tumor mutations and revealed the application potential of bile liquid biopsy technology in this challenging malignant tumor.

The cfDNA is primarily derived from normal leukocytes, stromal cells and tumor cells. Circulating tumor DNA (ctDNA) constitutes a small portion of cfDNA. It is usually actively secreted by tumor cells or released into the circulatory system during tumor cell apoptosis or necrosis. Mutations and methylation of ctDNA have been used as detection indicators in the early diagnosis of various malignancies.^22^, ^23^, ^24^, ^25^ However, the amount of cfDNA may be insufficient for reliable detection in early-stage tumors.^24^^,^^26^^,^^27^ Due to the natural anatomical advantage, bile cfDNA is primarily affected by hepatocytes and duct tissues, which are expected to contain a higher content of tumor-derived cfDNA than plasma in CCA patients.^28^ In this study, the concentration of tumor-derived DNA in bile demonstrated an overwhelming advantage over plasma, providing a solid biological basis for its diagnostic superiority. Consequently, the somatic mutation detection rate in bile was seven times higher than that in plasma, with significantly elevated variant allele frequencies (VAFs) reflecting enhanced characterization of tumor DNA. These findings fundamentally challenge the traditional “plasma-first” paradigm for liquid biopsy in biliary tract malignancies, establishing bile as an ideal biological sample for molecular testing.

Previous studies have mapped the genomic landscape of BTC and indicated that distinct CCA pathological subtypes may exhibit differences in treatment-relevant genotypes.^1^^,^^16^^,^^29^, ^30^, ^31^ Similar findings were observed in the present study. The high concordance of mutations between bile and tissue in this study demonstrated the exceptional capability of biliary cfDNA to recapitulate tumor heterogeneity. Notably, bile detected 47 out of 103 (45.6%) tissue variant sites, while plasma detected only 2 out of 103 (1.9%), including clinically significant *TP53* and *KRAS* mutations. This molecular-level integrity was further reflected in the comparable TMB between tissue and bile, whereas plasma failed to yield detectable TMB. In this study, our biliary sample identified a *BRAF* point mutation (p.V600E), which was present in both the tissue sample and biliary cfDNA, but absent in plasma cfDNA. These data suggest that this patient might benefit from the *BRAF* inhibitor Dabrafenib, and that biliary cfDNA may be a feasible tool for identifying patients eligible for targeted therapies. Molecular profiling of Western intrahepatic iCCA cohorts reveals *IDH1* genomic alterations in 13% of cases and *FGFR2* fusion events in 8%–14%.^32^, ^33^, ^34^ In this study, we detected an *FGFR2* fusion in both tumor tissue and matched bile samples, while it was undetectable in plasma cfDNA. This observation indicates that reliance on plasma cfDNA testing rather than bile cfDNA analysis would have failed to identify this patient as a candidate for pemigatinib therapy, an *FGFR2* potent inhibitor. Unexpectedly, no *IDH1/2* gene mutations were detected in the samples of this study, possibly due to the limited sample size.

Our data clarify the contextual limitations of plasma liquid biopsy technology. Although the detection rate of plasma cfDNA was significantly correlated with disease stage, bile cfDNA analysis consistently maintained high sensitivity and was unaffected by AJCC stage, lymph node status, or Child-Pugh grade. This stage-independent characteristic suggests that bile may overcome plasma's inherent limitations in detecting early-stage lesions. Current serum-based diagnostic tools for CCA, notably CEA and CA19-9 demonstrate suboptimal diagnostic performance due to variable sensitivity and specificity. Consequently, histopathological verification through invasive sampling remains the diagnostic benchmark.^35^ This study demonstrated that CA19-9 had a positive rate of 80% for CCA detection, while the combined use of CA19-9 and biliary cfDNA achieved a positive rate of 90%. This establishes an innovative clinical pathway for the synergistic diagnostic value of bile cfDNA and CA19-9. When standard biomarkers suggest malignant potential, biliary analysis can simultaneously confirm the diagnosis and enable molecular profiling, obviating the need for additional invasive procedures.

There are several limitations in our study. Although the results substantially align with larger-scale bile studies in advanced biliary tract cancers, we acknowledge that the primary limitation of this study is its small sample size (*n* = 10) and the sub-type analysis is also subject to the same constraints. As such, this work should be considered a pilot/feasibility study. Additionally, the inability to obtain bile samples from benign lesions for comparison due to ethical restrictions precludes the assessment of diagnostic specificity. Subsequent investigations will address this by analyzing cases of non-malignant biliary obstruction and establishing prospective multi-center cohorts for validation. Given the concentration advantage of bile cfDNA, its application in exploring minimal residual disease (MRD) monitoring holds greater promise than plasma-based approaches. Furthermore, extending the analysis to include bile-derived RNA and methylation markers may significantly enhance diagnostic sensitivity, aiding early screening efforts.

## 5 Conclusion

In conclusion, bile cfDNA demonstrated a significant advantage over plasma cfDNA in the somatic mutation detection of resectable CCA due to its lower susceptibility to the effects of tumor progression. In the future, bile cfDNA has the potential to serve as a reliable sample for NGS analysis, enabling early diagnosis and the post-operative tumor monitoring tool of CCA.

## CRediT authorship contribution statement

**Songyao Liu:** Writing – review & editing, Writing – original draft, Validation, Resources, Investigation, Formal analysis, Conceptualization. **Xiaowu Ma:** Writing – review & editing, Writing – original draft, Visualization, Validation, Resources, Formal analysis, Conceptualization. **Hongkai Zhuang:** Writing – review & editing, Validation, Resources, Methodology, Investigation, Conceptualization. **Bingkun Wang:** Writing – review & editing, Validation, Resources, Methodology, Formal analysis. **Qingbin Wang:** Writing – review & editing, Visualization, Resources, Methodology, Formal analysis. **Yonglin Hua:** Writing – review & editing, Visualization, Resources, Methodology, Formal analysis. **Ziyu Zhang:** Writing – review & editing, Visualization, Resources, Methodology. **Yuxin Xiao:** Writing – review & editing, Visualization, Resources, Methodology. **Jianbo Wan:** Resources, Writing – review & editing. **Yajin Chen:** Writing – review & editing, Validation, Supervision, Resources, Project administration, Funding acquisition, Formal analysis, Data curation, Conceptualization. **Changzhen Shang:** Writing – review & editing, Validation, Supervision, Resources, Project administration, Funding acquisition, Formal analysis, Data curation, Conceptualization.

## Informed consent

Informed consent was obtained from each patient for the use of their tumor, peripheral blood, and bile samples.

## Ethics statement

The study was approved by the Ethics Committee of Sun Yat-sen Memorial Hospital, Sun Yat-sen University (No. SYSKY-2023-043-01).

## Data availability statement

The data that support the conclusions of this study can be made available by the corresponding author upon reasonable request.

## Declaration of generative AI and AI-assisted technologies in the writing process

During the preparation of this work, the authors used Deepseek to improve the readability of the content. After using this tool, the authors reviewed and edited the content as needed and take full responsibility for the content of the publication.

## Funding

This research was funded by 10.13039/501100001809National Natural Science Foundation of China (82573407); 10.13039/501100021171Basic and Applied Basic Research Foundation of Guangdong Province (2023A1515220211); 10.13039/501100010256Guangzhou Municipal Science and Technology Project (2024B03J1335,2024A03J1129).

## Declaration of competing interest

The authors declare that they have no conflicts of interest. Yajin Chen is an Editorial Board Member of the journal, and he was not involved in the editorial review or the decision to publish this article.
